# Feasibility of SERS-Active Porous Ag Substrates for the Effective Detection of Pyrene in Water

**DOI:** 10.3390/s22072764

**Published:** 2022-04-03

**Authors:** Angela Capaccio, Antonio Sasso, Giulia Rusciano

**Affiliations:** 1 Department of Physics “E. Pancini”, University of Naples Federico II, 80126 Naples, Italy; antonio.sasso@unina.it (A.S.); giulia.rusciano@unina.it (G.R.); 2 National Research Council-National Institute of Optics (CNR-INO), 80078 Pozzuoli, Italy

**Keywords:** surface-enhanced Raman spectroscopy (SERS), Ag nanostructures, PAH detection, environment monitoring

## Abstract

Polycyclic aromatic hydrocarbons (PAHs) are ubiquitous pollutants that are typically released into the environment during the incomplete combustion of fossil fuels. Due to their relevant carcinogenicity, mutagenicity, and teratogenicity, it is urgent to develop sensitive and cost-effective strategies for monitoring them, especially in aqueous environments. Surface-enhanced Raman spectroscopy (SERS) can potentially be used as a reliable approach for this purpose, as it constitutes a valid alternative to traditional techniques, such as liquid and gas chromatography. Nevertheless, the development of an SERS-based platform for detection PAHs has so far been hindered by the poor adsorption of PAHs onto silver- and gold-based SERS-active substrates. To overcome this limitation, several research efforts have been directed towards the development of functionalized SERS substrates for the improvement of PAH adsorption. However, these strategies suffer from the interference that functionalizing molecules can produce in SERS detection. Herein, we demonstrate the feasibility of label-free detection of pyrene by using a highly porous 3D-SERS substrate produced by an inductively coupled plasma (ICP). Thanks to the coral-like nanopattern exhibited by our substrate, clear signals ascribable to pyrene molecules can be observed with a limit of detection of 23 nM. The observed performance can be attributed to the nanoporous character of our substrate, which combines a high density of hotspots and a certain capability of trapping molecules and favoring their adhesion to the Ag nanopattern. The obtained results demonstrate the potential of our substrates as a large-area, label-free SERS-based platform for chemical sensing and environmental control applications.

## 1. Introduction

The ever-increasing industrial development and human activities in the last years are leading to alarming atmospheric pollutant levels, and thus, the problem of their detection is at the center of scientists’ attention [1]. Among the organic airborne contaminants, polycyclic aromatic hydrocarbons (PAHs) are of great concern for both the environment and human health due to their carcinogenic and mutagenic properties. Unsurprisingly, the US Environmental Protection Agency (EPA) has classified 16 PAHs as high-priority pollutants. PAHs are composed of two or more fused benzene rings arranged in different structural configurations, but it is quite common to find PAH derivatives in environmental matrices that contain other atoms (nitrogen, oxygen, sulphur) or alkyl groups. They are primarily released into the atmosphere through the incomplete combustion of organic matter from both anthropogenic and natural emission sources, such as volcanic eruptions, forest fires, and fossil fuels [2,3,4]. Afterwards, the high hydrophobicity, the chemical stability, and the sorption capacity of these compounds contribute to their dispersion and persistence in soil, sediments, and water surfaces, making them ubiquitous in various worldwide locations [5,6,7]. As a matter of fact, according to the EU Water Framework Directive adopted in 2000, the maximum total levels of PAHs allowed in water should not exceed the value of 0.1 μg/L [8]. This recommendation boosted the development of novel techniques for the detection of ultra-low PAH levels in water environments.

Currently, the most common strategies for revealing PAHs in water samples are based on the combination of ultra-high liquid chromatography (UHLC) with fluorescence/ultraviolet detection (FLD/UV) [9,10,11]. In addition, gas chromatography coupled with flame ionization detection (GC-FID) or mass spectroscopy (GC-MS) is also used [12,13,14,15]. These techniques offer high accuracy and sensitivity, even allowing the detection of concentrations on the order of ng/L. However, the high cost and complex procedures required for the sample preparation make PAH analysis quite arduous and time-consuming. Alternatively, other low-cost, sensitive, and rapid approaches have been proposed to overcome the limitations of the conventional chromatographic techniques. Such strategies include biological methods [8,16], electrochemical sensors [17], and spectroscopic-based techniques [18].

By virtue of the label-free nature and molecular specificity of Raman spectroscopy, surface-enhanced Raman spectroscopy (SERS) has attracted great interest in the field of ultra-low revelation of chemical contaminants in water [19]. The ability of SERS to greatly intensify the Raman signals of bio-molecules bound to or near plasmonic nanostructures leads to an elevated sensitivity down to the single-molecule level [20]. This amplification is mainly due to the excitation of the localized surface plasmon resonances (LSPRs), which originate from the resonant collective oscillation of conduction electrons on noble metal surfaces with nanoscale features. The physical–chemical adsorption of a molecule on the metal nanostructure also gives rise to so-called chemical enhancement; this is primarily associated with charge-transfer transitions occurring between the electronic states of the metal–adsorbate surface complex [21]. The total enhancement factor (EF) resulting from the product of the two contributions can generally reach $10^{4}$–$10^{6}$ and can be even greater than $10^{10}$ in a few cases [22].

As reported in the literature, SERS has been successfully used to reveal traces of organic contaminants with a limit of detection (LOD) down to few ng/L [23]. In most cases, the SERS-active metal surface (generally Ag or Au) requires an appropriate chemical functionalization to adsorb the PAH molecules. The surfaces are generally modified by adding groups such as thiols, alkyl chains, antibodies, or β-cyclodextrin (β-CD). Other strategies are based on the immobilization of PAH molecules on graphene-based supporters, to which plasmonic nanoparticles are then added [24,25]. As a matter of fact, PAHs do not contain any functional groups (except in the case of derivates) that could favor the metal–adsorbate bonding; hence, the chemical enhancement with SERS [26]. However, if, on one hand, such superficial chemistry strategies can improve the selectivity of a specific compound for the SERS-active platform, on the other, they can reduce the sensitivity of SERS as long as the hotspots are occupied by the functional compounds. In addition, the portion of the SERS signal coming from these molecules, which are in close proximity to the substrate, may interfere with that from the upper layers, where the analyte lies (*first-layer effect*). Eventually, the intrinsic SERS spectra of the PAHs might be complicated, leading to an erroneous assignment of SERS bands [27].

Owing to these drawbacks, herein, we propose the detection of PAHs dissolved in water by using Ag-based nanoporous SERS substrates that do not require any surface chemical modifications. The proposed devices are prepared by following our recently reported procedure [28,29]. The substrates are characterized by a *coral-like* nanopattern that gives rise to an intricate porous silver network. We found that the porosity promotes the in-filtering of a PAH solution in the interstices and, thus, the “trapping” of the PAH molecules that end up being adsorbed on the metal surface through non-covalent interactions [30]. Moreover, the high enhancement factor of the order of $10^{7}$ and the broadband optical response in the Vis–NIR spectral range of our substrates enable the amplification of characteristic Raman features in a larger spectral range (200–4000 cm${}^{-1}$) that is not otherwise available. With such properties, the direct SERS detection of pyrene dissolved in water has been successfully achieved with an LOD of 23 nM.

## 2. Materials and Methods

### 2.1. Pyrene Solution Preparation

Pyrene was obtained from Sigma-Aldrich in powder form. Given the low solubility of pyrene in water (0.135 mg/L at 25 °C), the powder was firstly dissolved in methanol (≥99.9%, Sigma-Aldrich Co. Ltd., St. Louis, MI, USA) at a stock concentration of 1 μM and then serially diluted in Milli-Q water at concentrations ranging from 0.5 μM to 5 nM.

### 2.2. Fabrication and Characterization of SERS Substrates

**Figure 1:**
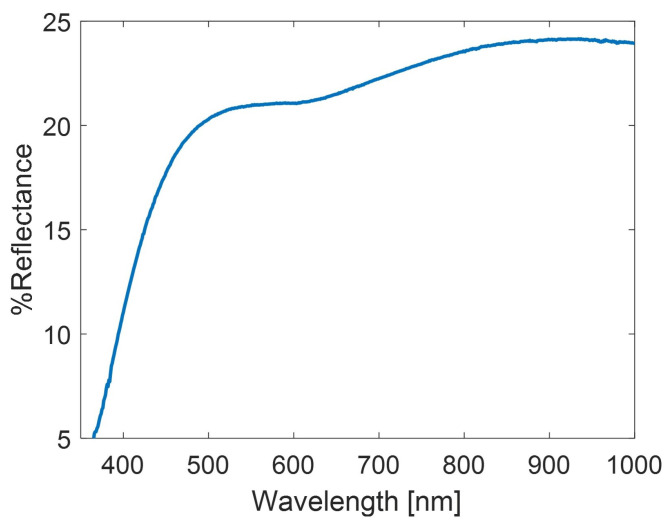
UV–Vis reflectance spectrum of the SERS substrate.

SERS substrates were prepared by following the procedure described in [28]. Briefly, a 3 nm Cr/10 nm Au bilayer was firstly sputtered (Q300T D Plus, Quorum Tech., Lewes, England) on a 15 × 15 mm${}^{2}$ clean glass coverslip, after which a 30 nm Ag film was deposited on the overlying Au layer. The deposition of the Cr/Au bilayer was essential for improving the adhesion of the overlying Ag layer during the successive plasma treatment. Therefore, in order to induce the formation of SERS-active Ag nanostructures, the Ag film was exposed to an inductively coupled plasma (ICP) treatment (PDC-32G-2, Harrick Plasma, Ithaca, NY, USA) for 90 s in a synthetic air atmosphere. As previously demonstrated through Raman and XRD measurements [28], this step led to the oxidation of the Ag film and the consequent loss of plasmonic activity. The pristine metallic character of the Ag layer was restored through a further 50 s of plasma treatment in an Ar atmosphere. The Ar-based plasma was able to reduce the AgO layer, preserving the former nanopatterning. The characteristic nano-texture gave rise a very broadband Vis–NIR optical response (Lambda 35, Perkin Elmer, Waltham, MA, USA), as shown in Figure 1. In this way, even Raman peaks lying in the near-IR spectral range can benefit from SERS amplification. The plasmonic activity of the SERS substrate was quantified using 4-MBA (99%, Sigma-Aldrich Co. Ltd.) as a probe molecule [28]. The outcome of this analysis revealed an average of EF∼$10^{7}$ with a high spatial reproducibility. The fluctuation of the SERS signal was evaluated by acquiring a series of raster scans, each consisting of 2500 spectra, randomly distributed over the whole substrate area *A* (A=2.25 cm${}^{2}$). Globally, we observed a percentage variability of scan-to-scan average SERS intensity of ∼7%, which assured a good signal stability and repeatability for quantitative SERS experiments.

The morphology of the substrate nanopattern was obtained with a field-emission scanning electron microscope system (SEM) (Merlin Compact VP, ZEISS, Oberkochen, Germany) operating at an accelerating voltage of 5 kV. The SEM analysis revealed that the average dimension of nanostructures, evaluated as the diameter of a circle of equal projection area $d_{EQPC}$, corresponded to ∼100 nm [28]. The SEM images were also used to evaluate the substrate porosity *p*, which was defined according to [31]. Briefly, starting from the grayscale SEM image, a binary mask was created by adjusting the lower- and upper-level thresholds. Therefore, *p* was evaluated with the ratio:

(1) $$p\left[\%\right]=\frac{\mathrm{black}\;\mathrm{pixels}\;\mathrm{number}}{\mathrm{total}\;\mathrm{pixels}}$$

where the numbers of pixels in the white and in the black areas were counted through the built-in Matlab function *bwarea*.

### 2.3. Raman Setup

The Raman and SERS measurements were collected with a WiTec Alpha 300 confocal microscope equipped with a frequency-doubled Nd/YAG laser emitting at 532 nm as a Raman probe. Scattered photons were collected in a back-scattering geometry through a 20× dry objective lens and sent via a 100 μm core fiber to a 600 g/mm grating of a high-throughput spectrometer. Spectra were acquired with a thermoelectrically cooled CCD camera (T=−60 °C). The acquired spectra were processed by using tools available in the WiTec Project program. In particular, the acquired spectra were first processed in order to remove the contribution of cosmic rays; therefore, a fourth-order polynomial background was removed. Finally, a Savitsky–Golay filter was applied for denoising of the spectra.

### 2.4. SERS Measurements

**Figure 2:**
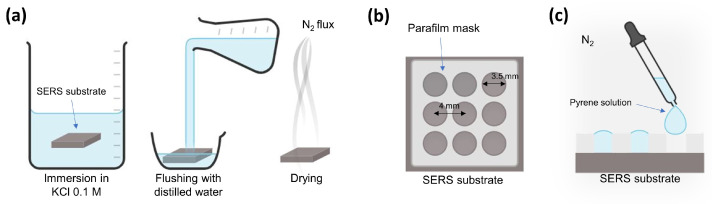
Scheme of the preparation of the SERS substrate and sample deposition for SERS measurements. (**a**) Cleaning and drying of the SERS substrate; (**b**) parafilm mask composed of nine wells glued on the *clean* SERS platform; (**c**) dripping of the pyrene solution into the wells and drying in N${}_{2}$ atmosphere.

The spectra of pyrene at different concentrations were analyzed on the same substrate in order to reduce the fluctuations in the SERS signals’ intensities due to the inter-batch reproducibility of the substrates. The preparation of the SERS substrates for the measurements is schematized in Figure 2. Firstly, the as-prepared substrate was immersed in a 0.1 M KCl solution for 20 min, followed by abundant rinsing with distilled water and drying with a gentle N${}_{2}$ flux (part a). This method was effective in removing the residual contaminants that were eventually adsorbed in the silver nanopattern [32]. After that, a parafilm (Bemis Parafilm “M” Laboratory Film) mask exhibiting 9 wells (each 3.5 mm in diameter) was prepared and thermally glued to the freshly prepared SERS substrate (part b). The wells were filled then with 20 μL of pyrene solution at different concentrations and allowed to dry at room temperature in a controlled N${}_{2}$ atmosphere (part c).

## 3. Results and Discussion

### 3.1. Analysis of the SERS Substrate Porosity

Substrate porosity plays a key role in the SERS-based detection of PAHs. As a matter of fact, the employment of porous metallic structures can be an interesting approach for trapping molecules that lack in surface affinity to metallic surfaces [33]. Compared to the strategies that include the functionalization of a metal surface to favor the binding of aromatic molecules [34], three-dimensional (3D) SERS substrates promote the capture of PAHs that adhere to the Ag surface through non-covalent interactions, thereby avoiding the *first-layer effect* that occurs for functionalized SERS substrates [27]. In addition, the intrinsic 3D character of the porous structure offers a larger surface area for target molecule exposure [35], which extends the exploitable concentration range for SERS analysis.

**Figure 3:**
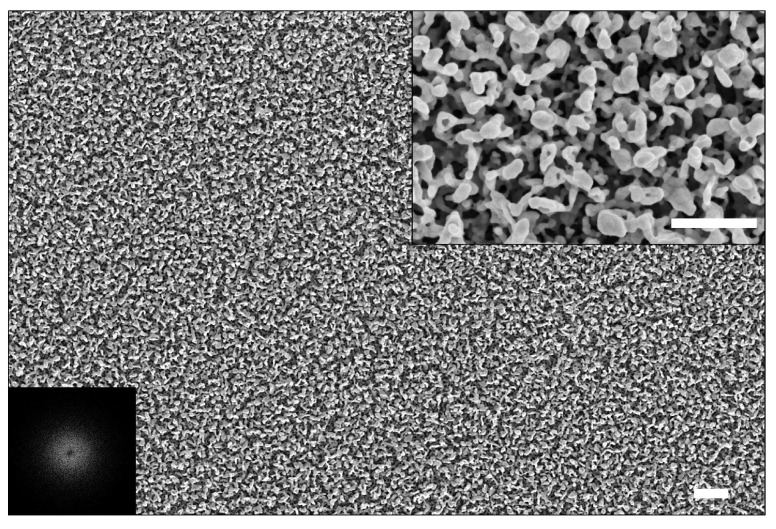
SEM image of a *coral-like* substrate (scale bar = 1 μm). The inset in the upper-right corner shows the magnification of the SEM image (scale bar = 500 nm), whereas the inset in the lower-left corner shows the PSD.

The porosity of our substrates was estimated by starting from scanning electron microscope (SEM) images. Figure 3 shows a typical SEM image of an SERS substrate prepared by following the procedure described in the Materials and Methods section. As it is possible to see, the obtained silver nanopattern exhibits an intricate network of nanocavities, which act as cages for PAHs. The substrate porosity was quantitatively estimated according to the approach described in the Materials and Methods section. For the substrate shown in Figure 3, we estimated a porosity equal to 54%.

Notably, the substrate nanopattern is quite uniform on the microscale, which assures a good signal stability by collecting the signal on the beam waist of a 20× objective lens (∼1.4 μm${}^{2}$ area). On the other hand, the isotropy of the nanopattern is revealed by the radial symmetry of the power spectral density (PSD), which is shown in the lower-left corner of Figure 3, whereas the ring shape highlights a long-range order of the observed morphological features [36,37,38].

### 3.2. Assessment of Pyrene Detection

**Figure 4:**
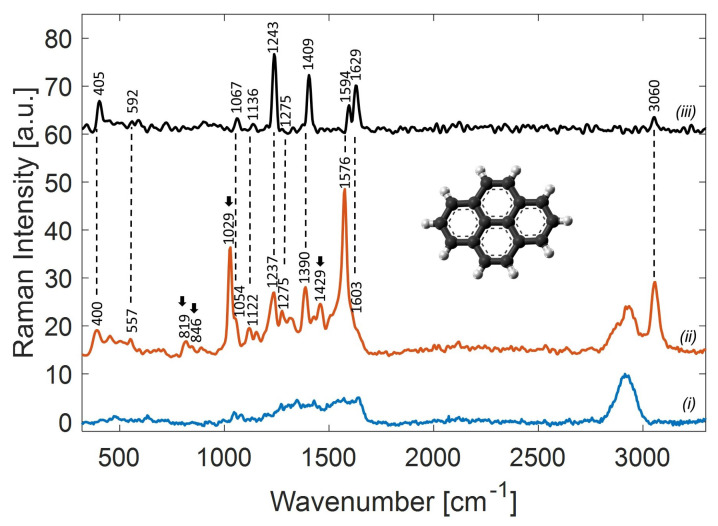
Comparison of (i) the SERS spectrum of the solvent (water) control acquired on a *clean* SERS substrate after the evaporation of a distilled water droplet and (ii) the SERS spectrum of a 1 μM pyrene solution. The arrows indicate the presence of further peaks in the SERS spectrum. The Raman spectrum of solid pyrene is also shown (iii) and rescaled with respect to the spectra (i) and (ii) to assure a better visualization.

As described above, the detection of PAHs with SERS is hindered by their very low affinity for metallic surfaces, which intrinsically reduces the observable SERS signal for this kind of molecule. As a matter of fact, the number of PAH molecules in the hotspots can be very low, and the resulting SERS signals can be hidden by noise. Furthermore, molecules that contribute to air impurity may occupy hotspots, especially if their surface affinity is higher than that of PAHs. In this condition, the signal from weakly adsorbed PAHs can totally disappear, even at concentrations in the micromolar range. For this reason, particular care was taken in order to reduce the interference of contaminants during SERS-based pyrene detection. Figure 4, trace (i), reports the SERS spectrum of the solvent (water) control, which was obtained after the evaporation of distilled water on a *clean* substrate. The residual background signal exhibits a distinguishable contribution around 3000 cm${}^{-1}$, which can reasonably be thought to be due to organic contaminants. Instead, the fingerprint region (400–2000 cm${}^{-1}$) is relatively free from contaminants, showing only broad residual features originating from C-C and C-H bonds in different environments. Once the potential interference from contaminant molecules was assessed, we proceeded by analyzing the potentiality of our substrates for pyrene detection. For this purpose, a 20 μL drop of 1 μM pyrene solution was dripped into one of the substrate wells and allowed to dry in a controlled N${}_{2}$ atmosphere. In this condition, the molecular surface density (molecules in the apparent surface area) of ∼10${}^{6}$ mol/μm${}^{2}$ could be estimated. Figure 4, trace (ii), reports the SERS signal acquired as trace (i) by using a Raman probe power of $P_{R}=180$ μW and an integration time of τ=1 s. For comparison, Figure 4, trace (iii), shows the Raman spectrum of solid pyrene, obtained at $P_{R}$ = 1.5 mW and τ = 2 s.

A detailed assignment of the peaks observed for both Raman and SERS spectra is reported in Table 1. Notably, the observed Raman spectrum is in reasonable agreement with the literature [39]. Through comparative analysis, we observed that all Raman peaks, except at 1275 and 3060 cm${}^{-1}$, undergo a red-shift in the corresponding SERS spectrum. This variation is consistent with the formation of the metal–adsorbate complex, which leads to a redistribution of the electronic cloud and, hence, to a change in polarizability. As reported in [40], when the transfer of electrons from metal to PAH molecules increases via the charge-transfer mechanism, the Raman peaks shift downwards to lower frequencies. In addition, Wan et al. [41] found that the change in the interatomic bond force and length contributes to the red shift of Raman peaks. Finally, the fingerprint region of the SERS spectrum shows the presence of further peaks (indicated by arrows in Figure 4) that are not observable in the spontaneous Raman spectrum. Such behavior is very common in SERS spectra [42]. In fact, based on SERS selection rules, the bands relative to vibrational modes in the perpendicular direction with respect to the metal surface can be selectively amplified. Moreover, the local field enhancement in the hotspot is also strongly dependent on the coupling between the hotspot and the external excitation [43]. In this way, weak vibrational modes that are not visible in the relative Raman spectrum can also be detected thanks to the enhancement provided by the SERS technique.

Beyond the fingerprint region that was analyzed so far, it is worth noting that even the bands lying in the 2500–4000 cm${}^{-1}$ range are amplified via the effect of SERS. This amplification is due to the broad plasmon resonance of our SERS substrate, which covers both the visible and NIR regions, as shown in Figure 1. In fact, the variety in shapes and dimensions of our 3D Ag nanostructures can produce not only a high density of hotspots, but also optical resonances. Both the inter-particle coupling [44] and the high shape factor of the NPs [45] can produce multipolar resonances that contribute to the final overlapping plasmon response. Thanks to this property, it is possible to extend the plasmon amplification quite far from the laser probe wavelength, also reaching the peak at 3060 cm${}^{-1}$. In this way, we can monitor the abundance of pyrene by using this peak, which, to our knowledge, has never been used before for SERS-based pyrene detection.

### 3.3. Calibration Curve and Limit of Detection (LOD)

**Figure 5:**
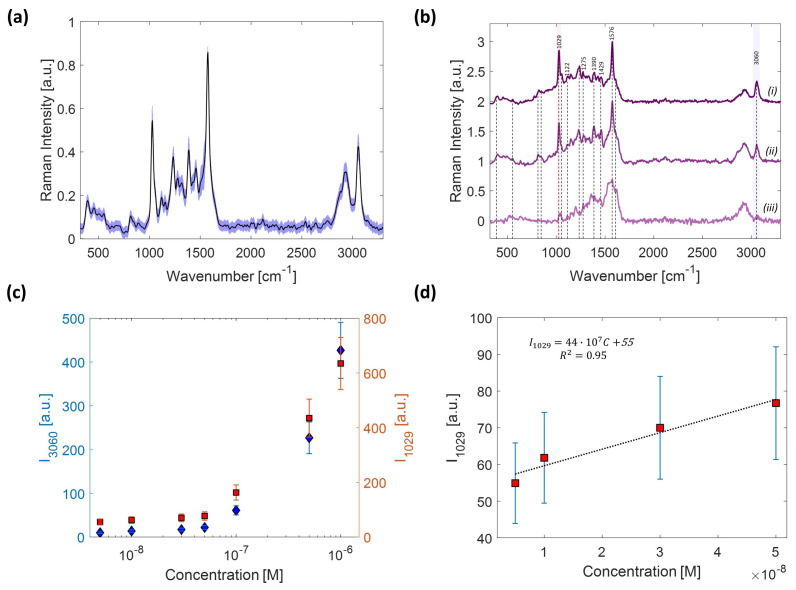
(**a**) SERS spectra of a 1 μM pyrene solution from a set of five measurements that were randomly carried out over the surface of the well area $A_{w}$. The shaded blue area shows the variability of SERS signals corresponding to ∼7%; (**b**) comparison of the average SERS spectra of pyrene acquired at 0.5 μM (i), 0.05 μM (ii), and 5 nM (iii); (**c**) plot of the SERS peak intensity at 1029 cm${}^{-1}$ (red) and 3060 cm${}^{-1}$ (blue) versus the concentration of the pyrene solution; (**d**) linear trend of the signal at 1029 cm${}^{-1}$ in the lower concentration range. The best-fit line is also reported, together with the line’s equation.

As the last step, the sensitivity for pyrene detection in an aqueous environment when using our 3D nanoporous substrate was quantitatively investigated. For this purpose, we performed SERS measurements on pyrene samples at different concentrations, ranging from 1 μM to 5 nM, while following the procedure that was previously described. For each sample, SERS spectra were collected with five raster scans at different points of the substrate well. Every scan was performed in a 25×25 μm${}^{2}$ area for a total of 100 spectra ($P_{R}$ = 180 μm, τ = 1 s). Figure 5a shows the SERS spectra coming from the five measurements for the pyrene solution at 1 μM. The shaded area highlighted in blue is due to the overlapping of SERS signals that, globally, show a variability of ∼7%, which is in agreement with our previous study [28]. This result demonstrates the high reproducibility of our SERS substrates over the whole area of a single well $A_{w}$ ($A_{w}$∼10 mm${}^{2}$). Figure 5b shows the average SERS spectra obtained at (i) 0.5 μM, (ii) 0.05 μM, and (iii) 5 nM concentrations. The spectra were normalized at the prominent peak at 1576 cm${}^{-1}$ and vertically shifted for a better visualization. The dashed vertical lines highlight the presence of spectral features due to pyrene. Notably, the peaks at 1029, 1122, 1275, 1390, 1429, 1567, and 3060 cm${}^{-1}$ can still be appreciated, even at the lowest pyrene concentration. However, at the lowest concentration, the relative weight of the residual background signal in the fingerprint region becomes important, partially masking the underlying SERS signal of pyrene. Figure 5c shows the concentration-dependent SERS intensity relative to the peaks at 1029 and 3060 cm${}^{-1}$. The error bars are the standard deviation resulting from the SERS measurements in the different scans mentioned before. As shown, the intensity of each peak decreases in a similar fashion. In particular, two different and almost linear trends can be appreciated, depending on the concentration range. This behavior has been previously observed in SERS-based detection of PAHs [47].

The linear relationship found in the range between $0.5\times 10^{-7}$ and $0.5\times 10^{-9}$ M was used to estimate the limit of detection (LOD), which is defined as the lowest concentration at which the SERS signal can be discriminated from the background noise. The LOD is calculated according to the following formula [48]:

(2) $$\mathrm{LOD}=\frac{3\sigma_{y}}{b}$$

where $\sigma_{y}$ is the residual standard deviation and *b* is the slope of the regression line, which is shown in Figure 5d relative to the peak at 1029 cm${}^{-1}$, which offers a higher contrast with respect to the background. In this case, the estimated LOD of pyrene is 23 nM, which is an excellent result considering the higher LOD of pyrene (10 μg/mL) that has been found for SERS-active metal surfaces in the absence of surface functionalization [42]. In most cases, LODs of the order of ng/L are only achieved on metal surfaces that have been appropriately chemically modified to increase the binding affinity. On the contrary, our findings demonstrate that the intrinsic nanoporosity of our *coral-like* substrates can be enough to capture the organic molecules. In such way, a rather high sensitivity is achieved, avoiding the need for functionalization, which greatly spreads the range of applicability, holding promise for effective use in integrated SERS-based platforms for environmental monitoring.

## 4. Conclusions

In the current study, we presented a novel SERS-based approach for the detection of pyrene at the trace level in an aqueous solution. The protocol developed here for the nano-fabrication of SERS substrates allows one to obtain a uniform network of Ag nanopores that act as hotspots for the target molecules. Compared to the common practice of functionalizing SERS-active metal surfaces to favor the binding of PAH molecules, the coral-like substrates do not need any further modifications. In fact, our findings demonstrate an efficient detection of the SERS features of pyrene with an LOD of 23 nM. This confirms that porosity plays a dominant role in the adsorption of the molecules. From this perspective, the proposed SERS substrates could be used for the monitoring of organic contaminants in both liquid and gaseous environments. In addition, the proposed protocol for the development of porous nanostructures could potentially be applied to optical fiber sensors, paving the way for on-site applications.

## Figures and Tables

**Table 1:** Band assignments for Raman and SERS spectra of pyrene [42,46]. Note: δ, bending; ν, stretching.

Raman	SERS	Vibrational Modes
405	400	δ(CH) in-plane
592	557	Ring breathing
-	819	δ(CH) out-plane
-	846	δ(CH) out-plane
-	1029	δ(CH) in-plane
1067	1054	δ(CH) in-plane
1136	1122	δ(CH) in-plane, δ(CC) in-plane
1243	1237	δ(CH) in-plane
1275	1275	δ(CH) in-plane, δ(CC) in-plane
1409	1390	δ(CH) in-plane, δ(CC) in-plane
-	1429	Aromatic ν(CC)
1594	1576	δ(CH) in-plane, Aromatic ν(CC)
1629	1603	δ(CH) in-plane, Aromatic ν(CC)
3060	3060	Aromatic ν(CH)

## Data Availability

Not applicable.

## Abbreviations

The following abbreviations are used in this manuscript:

PAH	Polycyclic Aromatic Hydrocarbon
SERS	Surface Enhanced Raman Spectroscopy
LOD	Limit Of Detection
EF	Enhancement Factor
PSD	Power Spectral Density
NPs	Nanoparticles
